# Compressive dual-comb spectroscopy

**DOI:** 10.1038/s41598-021-93005-1

**Published:** 2021-06-29

**Authors:** Akira Kawai, Takahiro Kageyama, Ryoichi Horisaki, Takuro Ideguchi

**Affiliations:** 1 Department of Physics, The University of Tokyo, Tokyo, Japan; 2 Graduate School of Information Science and Technology, The University of Tokyo, Tokyo, Japan; 3 PRESTO, Japan Science and Technology Agency, Saitama, Japan; 4 Institute for Photon Science and Technology, The University of Tokyo, Tokyo, Japan

**Keywords:** Infrared spectroscopy, Frequency combs

## Abstract

Broadband, high resolution, and rapid measurements of dual-comb spectroscopy (DCS) generate a large amount of data stream. We numerically demonstrate significant data compression of DCS spectra by using a compressive sensing technique. Our numerical simulation shows a compression rate of more than 100 with a 3% error in mole fraction estimation of mid-infrared (MIR) DCS of two molecular species in a broadband (~ 30 THz) and high resolution (~ 115 MHz) condition. We also numerically demonstrate a massively parallel MIR DCS spectrum of 10 different molecular species can be reconstructed with a compression rate of 10.5 with a transmittance error of 0.003 from the original spectrum.

## Introduction

In the last decade, intensive attention has been cast on dual-comb spectroscopy (DCS), which allows one to measure broadband and high-resolution spectra with superior frequency accuracy at a high data acquisition rate^1–3^. DCS provides outstanding spectroscopic features, especially for multiplex gas-phase molecular sensing with its high spectral resolution of ~ 100 MHz spanning over ~ 10 s THz, enabling various applications such as precision metrology^4^, greenhouse gas sensing^5,6^, combustion diagnosis^7^, etc. The broadband and high-resolution spectroscopy can generate a large data set, e.g., 1,000,000 spectral points for a single spectrum^8^. Now, if we imagine the DCS techniques are to be used for hyperspectral imaging of 1000 × 1000 pixels measured with a 16-bit analog-to-digital converter, it generates ~ 4 TB per single hyperspectral image. Taking such images would cause severe problems in data transportation and storage.

Compressive sensing (CS) is a signal processing technique that allows, by making use of sparsity of a signal, reconstruction of the signal from a significantly reduced number of data points than the full set of data points required from the Nyquist-Shannon sampling theorem^9^. If the signal is sparse on a certain basis, the sparsest solution can be found by algorithms with a sparsity constrain or regularization. Mathematical studies proved that, in some appropriate conditions, CS could reconstruct an exact signal even in the presence of measurement noise^10^. A variety of studies on CS have been reported especially in the field of optical imaging, where natural scenes such as landscapes or biological cells are well reconstructed from images with fewer pixels^10–12^. Contrary to imaging, CS-based spectroscopy^13–18^, especially for gas-phase molecular sensing, has not actively been investigated, although high-resolution broadband spectra of gaseous molecules are good candidates of CS because of their sparse nature originating from the narrow molecular lines spread in a broad spectral range.

In this study, we numerically demonstrate compressive dual-comb spectroscopy (C-DCS), in which a simple CS technique effectively compresses the data size of DCS. In our numerical demonstration, we show that quantitative estimation of mole fraction of molecules can be made with an error of 3% even when < 1% of interferometric data points are used only. Also, we show a well-reconstructed complex spectrum of a mixture of 10 molecular species in the condition of using 10% of original data points.

## Results

### Concept of compressive dual-comb spectroscopy (C-DCS)

**Figure 1:**
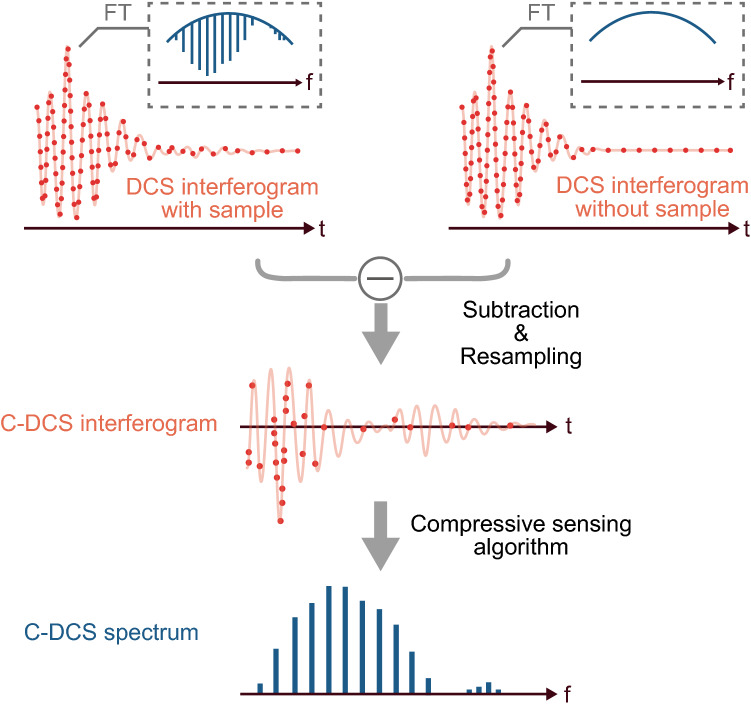
A conceptual illustration of compressive dual-comb spectroscopy (C-DCS).

The basic concept of C-DCS is described in Fig. 1. DCS is a type of Fourier-transform spectroscopy with two mutually coherent frequency combs that run at slightly detuned repetition rates^1–3^. The spatially combined pulse trains are interferometrically detected with a single photodetector, and digitization of the signal generates an interferogram. Finally, Fourier-transforming of the interferogram shows a spectrum. Our simulation is conducted under the assumption that we have a complete original data set of a DCS interferogram and randomly resample the data points at a lower rate (reducing the data points) to reconstruct a CS spectrum. Note that the random sampling guarantees decorrelation of the measurement data and the sparse basis (Fourier basis, in this case), which is a requirement of efficient CS^9^. It also ensures the generality of CS that works for any spectral shapes (e.g., condensed or spread absorption lines) when the sparsity is at the same level. Suppose that the number of data points of the original interferogram is $N$ and that of the resampled data points is $M$ ($M<N$), the resampled data set can be represented as ${\Omega }=\{{\omega }_{j}{\}}_{j=1}^{M}\subset \{\mathrm{1,2},\dots ,N\}$. Note that we can arbitrarily set a probability mass function (PMF) for the random resampling. From the resampled data set, we can estimate a spectrum vector $x \in {\mathbb{C}}^{N}$ as an answer to the *l*1 minimization problem^9^ described as Eq. (1),

1 $$\mathrm{min} \, \big|\big|\stackrel{\sim }{x}\big|\big|_{1}   \, {\text{subject}} \, {\text{to}} \, \big|\big|\mathrm{A}\stackrel{\sim }{x}-y \big|\big|_{2}< \epsilon $$

where $\mathrm{A}\in {\mathbb{C}}^{M\times N}$ ($M<N$) is a sensing matrix, $y\in {\mathbb{R}}^{M}$ a measurement vector, and $\epsilon \in \mathbb{R}$ a constraint value determined by noise of the system. In our case, $\mathrm{A}=\mathrm{R}\mathrm{\Phi }$, where $\mathrm{R}\in {\left\{0,1\right\}}^{M\times N}$ is a subsampling operator which obeys $(\mathrm{R}x{)}_{j}={x}_{{\omega }_{j}}$, and $\mathrm{\Phi }$ a $N\times N$ discrete Fourier-transform operator. Considering a case of DCS operated with relatively low-chirped combs, signal intensities of the sampled points around the zero delay between the pulses have a larger magnitude (called “center-burst”) than the other points, including the signals showing molecular induction decays^19^. Therefore, it is effective to select a sloped PMF that samples more points around the center burst.

To fully utilize the sparse nature of the absorption spectrum and efficiently compress the data points, we suggest operating background subtraction of the interferogram. It can be implemented either by hardware instrumentation or post numerical processing. For the hardware instrumentation, a Michelson-type interferometer is added to make the π phase difference between the pulses from the two arms due to the reflection of the beam splitter, realizing the background subtraction due to the destructive interference on the detector^20^. On the other hand, for the post numerical processing of background subtraction, a reference background interferogram can be obtained either by an additional measurement^21^ or a numerical baseline reconstruction^5^. Although in this proof-of-concept demonstration we operate the background subtraction for better reconstruction, we expect improved algorithms would make it possible to reconstruct spectra with a background.

### Numerical condition of C-DCS simulation

To show the above-mentioned C-DCS concept, we demonstrate numerical simulations of trace-gas DCS in the MIR region. We simulate a mimic condition of a previously reported experiment^21^, where a broadband spectrum covering from 2006.7 to 3013.4 cm^−1^ (60.159–90.339 THz) is measured at a resolution of 0.0038 cm^−1^ (115 MHz) that consists of 262,144 spectral points Fourier-transformed by temporal data points of 524,286. We assume a broadband Gaussian-profile spectrum as comb sources. We first simulate an interferogram from the source spectrum with molecular absorptions with Doppler line profiles and create a background-free interferogram by baseline subtraction with a reference spectrum with no absorption lines. We numerically calculate the spectrum by referencing the HITRAN database and using its application programming interface HAPI^22^. Then, the interferogram is resampled with a sloped PMF, $C \, \mathrm{min} \, \{1, 1/ | \, l -N/2 | \}$, where $l$ is an index of sampling points of the interferogram in chronological order and $C$ a normalization constant, which is found in the literature^23^. For spectrum reconstruction, we use SPGL1 as an *l*1-minimization problem solver^24^. We set an arbitral signal-to-noise ratio (SNR) by assuming coherently averaged interferograms, which can be experimentally implemented with a variety of techniques^8,25,26^. We note that the CS reconstruction algorithm, in general, works without having pre-knowledge of the molecular species.

### Mole fraction estimation of two molecular species

**Figure 2:**
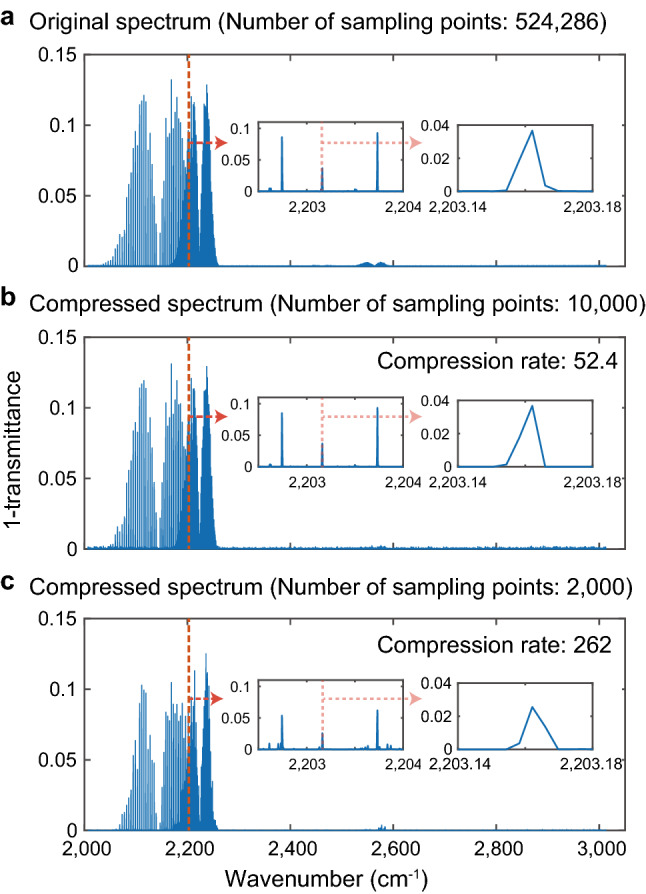
Simulated MIR DCS spectra of gaseous molecules. (**a**) An original spectrum Fourier-transformed by temporal data points of 524,286. (**b**, **c**) Compressed spectra with 10,000 and 2000 sampling points, respectively. The inset panels show zoom-in spectra around 2203 cm^−1^.

**Figure 3:**
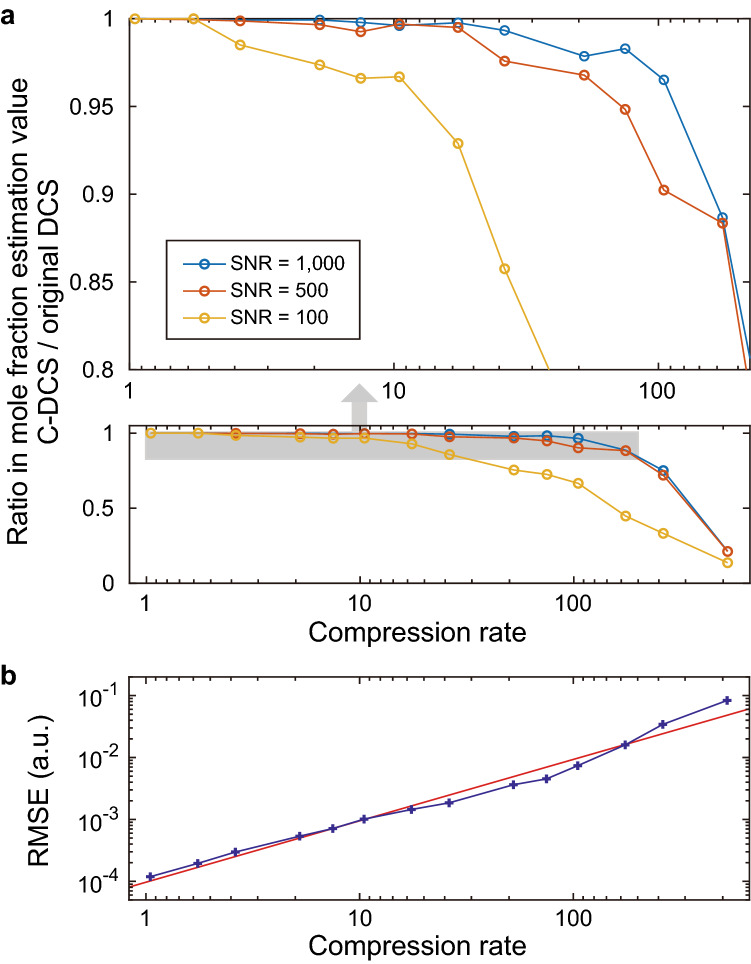
Mole fraction estimation from simulated spectra. (**a**) Ratio in mole fraction estimation value of C-DCS and original DCS with different compression rates. Blue, Red, and Yellow data show the results with different SNR. (**b**) Root-mean-squared error (RMSE) of the spectral points for each compression rate. The fitting (red line) shows that RMSE is proportional to (compression rate)^0.93^.

To show how the data compression rate, which is defined as $N/M$, affects the quantitative capability of C-DCS, we demonstrate mole fraction estimation of two molecular species of trace gases. We numerically prepare mixed gases of N_2_O (42 ppm) and CO (120 ppm) with a buffer gas at a pressure of 3 mbar filled in a 10-m-long multi-pass cell. We add a Gaussian measurement noise $\boldsymbol{n}$ to the interferogram to make the estimation with different SNR conditions. We first set the SNR of the real part of FFT to be 1000. A constraint term $\epsilon$ in Eq. (1) is empirically set to an average of $||\boldsymbol{n}/10||$. The original spectrum converted from the full data points of 524,286, and compressive spectra with 10,000 and 2000 sampling points are shown in Fig. 2, where we show transmittance spectra of the sample. The compressive spectrum with 10,000 sampling points, which compression rate is 52.4, shows good agreement with the original one, while that with 2000 (compression rate of 262) shows clear distortions. We evaluate the mole fraction of N_2_O molecules by spectrally fitting each absorption line and obeying Lambert–Beer law. The fitting is operated by a fixed-profile Gaussian function with a single free parameter of mole fraction. Here, the spectral points that satisfy $\mathrm{log}(1-\mathrm{T})>0.01$ ($\mathrm{T}$: transmittance) are used for the evaluation. Figure 3a shows the ratio of the evaluated mole fraction of the compressed and original spectra for different compression rates under different SNR (1000, 500, 100) conditions. The result with SNR of 1000 shows that the compression rate of 105, which corresponds to the number of sampling points of 5000, leads to a 3% of deviation of mole fraction from that evaluated with the original spectrum. We can also see that lower measurement SNR degrades the evaluation results. Figure 3b shows root-mean-squared error (RMSE) of the spectral points that satisfy $\mathrm{log}(1-\mathrm{T})>0.01$ for each compression rate. We find that RMSE is proportional to (compression rate)^0.93^ by least-squares fitting.

### Robustness evaluation of C-DCS reconstruction

**Figure 4:**
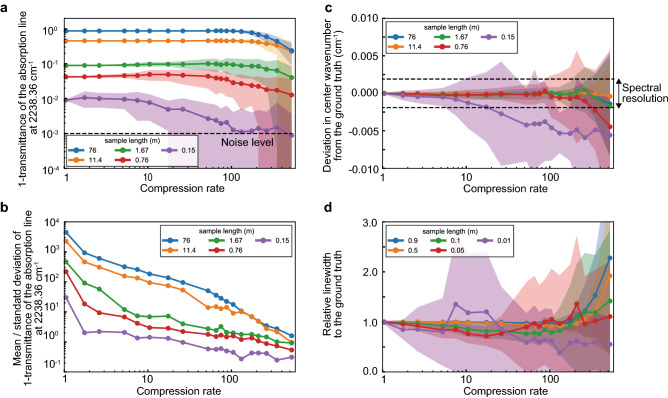
Robustness evaluation of CS reconstruction as a function of the compression rate. (**a**) peak transmittance. (**b**) mean/standard deviation of peak transmittance. (**c**) deviation in center wavenumber from the ground truth. (**d**) relative linewidth to the ground truth, of the absorption line at 2238.36 cm^−1^. The plots with different colors show the calculation results with different sample lengths (blue: 76 m, yellow: 11.4 m, green: 1.67 m, red: 0.76 m, purple: 0.15 m). The dots and bands represent the mean values and standard deviations of the 100 spectra calculated with the different random samplings.

We quantitatively evaluate the robustness (deviation from the ground truth) of the CS reconstruction in terms of the peak transmittance, center frequency, and linewidth of an absorption line by simulating spectra with 100 different patterns of random sampling for each condition. We analyze a single absorption line of N_2_O at 2238.36 cm^−1^ in the spectra calculated in the same condition as that shown in Fig. 2 with the SNR of 1000. Here we change the sample lengths (76, 11.4, 1.67, 0.76, 0.15 m) so that we can see how the absorption peak transmittance affects the CS reconstruction quality. Figure 4a shows the peak transmittance of the absorption line as a function of the compression rate. The standard deviations of the 100 spectra calculated with the different random samplings are illustrated as weak color bands around the mean values. Figure 4b shows the mean value divided by the standard deviation of the data points shown in Fig. 4a. It clearly shows the reconstruction degrades at higher compression rates, and the absorption lines with higher absorption intensities (longer sample lengths) are reconstructed more robustly. Figure 4c shows the deviation in the center wavenumber of the absorption line from the ground truth. The mean values are mostly within the spectral resolution (0.0038 cm^−1^), showing its high robustness in the CS reconstruction. Figure 4d shows the relative linewidth to the ground truth. Although the absorption lines with higher peak intensities are well reconstructed and no resolution degradation is observed up to the compression rate of ~ 100, the ones with lower peak intensities largely deviate from the ground truth at the higher compression rates.

### Massively parallel C-DCS of 10 trace gas species

**Figure 5:**
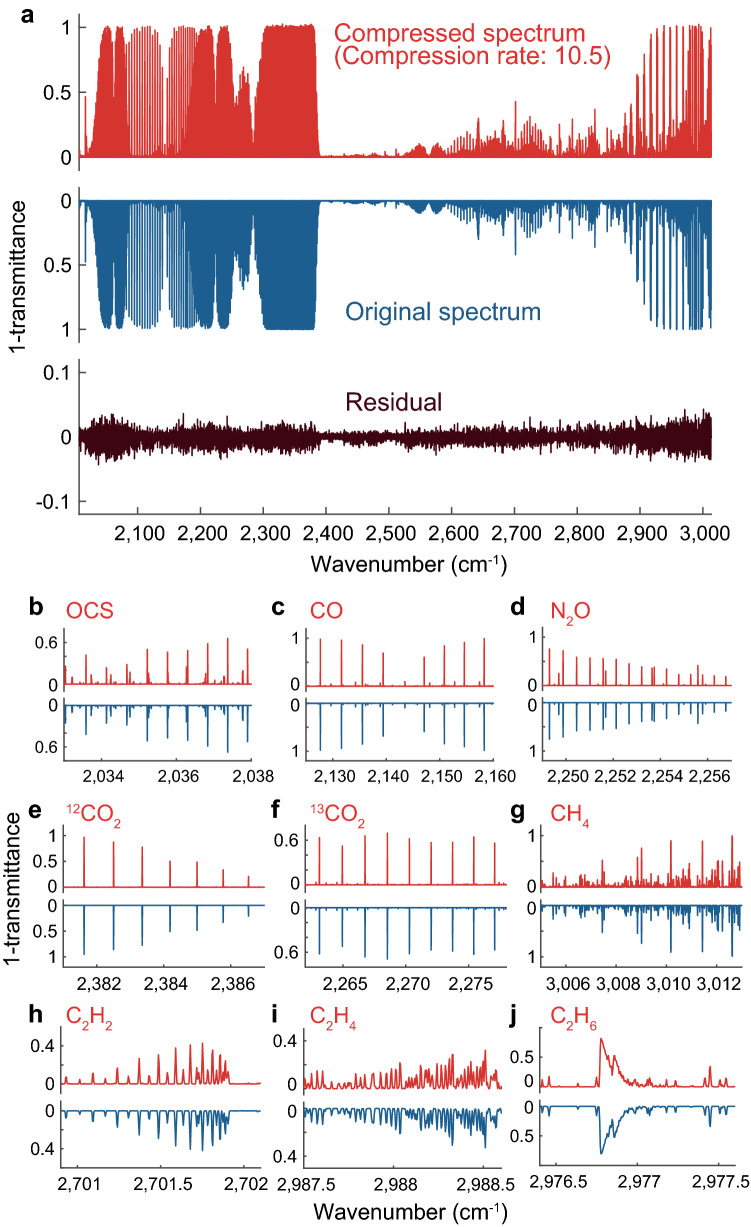
Massively parallel spectra of 10 trace gas species. (**a**) Comparison between a compressed spectrum and an original spectrum. The compressed spectrum is reconstructed from 50,000 points (compression rate of 10.5). (**b**–**j**) Zoom-in spectra of characteristic absorption lines of different molecular species.

Finally, to show the compression capability of C-DCS for denser molecular lines, we demonstrate massively parallel spectroscopy of 10 trace gas species, which resembles the previously reported experiment^21^. We assume a 76-m-long multi-pass gas cell filled with nitrous oxide (^14^N_2_^16^O) at 42 ppm, nitric oxide (^14^N^16^O) at 420 ppm, carbon monoxide (^12^C^16^O) at 120 ppm, carbonyl sulfide (^16^O^12^C ^32^S) at 26 ppm, methane (^12^CH_4_) at 1,500 ppm, ethane (^12^C_2_H_6_) at 490 ppm, ethylene (^12^C_2_H_4_) at 540 ppm, acetylene (^12^C_2_H_2_) at 6,600 ppm, carbon dioxide (^12^CO_2_, ^13^CO_2_), at 280 ppm, water vapor (H_2_^16^O) at 2,100 ppm and a buffer gas. The most abundant isotope of each element except for CO_2_ is included in this simulation. The pressure is set to 3 mbar. To relax regularization and let small absorption peaks remain, we increase the constraint term $\epsilon$ to the average of $||\boldsymbol{n}||$. Figure 5 shows the C-DCS spectra with a compression rate of 10.5. We observe that vibrational absorption lines are well reconstructed compared to the original ones with the error (standard deviation) in transmittance of less than 0.003.

## Discussion

The concept of C-DCS can apply to other Fourier-transform spectroscopy (FTS), including Michelson-type FTS, FT-Raman spectroscopy^27^, and FT-CARS spectroscopy^28,29^, etc. It can also be used for a spectrum measured in the spectral domain by calculating an interferogram by Fourier-transforming the spectrum. We point out that the CS is, in particular, useful for DCS because it generates a large amount of data with large spectral bandwidth and high spectral resolution, which ensures the most significant sparsity hence the efficient data compression. Also, the high scan rate of DCS generates a high-speed data stream that would cause foreseeable problems of data transportation and storage.

The performance of C-DCS can be improved by using other PMFs and/or reconstruction algorithms specifically developed for the use of CS imaging. The compression of C-DCS would become more valuable when we use it for higher dimensional measurements such as hyperspectral imaging^30^ or multi-dimensional DCS^31^. Lastly, we note that C-DCS is possibly used for speeding up DCS measurement by implementing the compressive sampling in hardware. For that purpose, for example, we can arbitrarily sweep the difference in repetition rate during measurement of an interferogram, allowing a non-uniform temporal waveform sampling.

## Conclusion

We proposed compressive dual-comb spectroscopy (C-DCS) to address the data size problem of high-speed, broadband, and high-resolution dual-comb spectroscopy. Our numerical demonstration of C-DCS of the two molecular species (N_2_O and CO) showed the reduction of the required data points by more than a factor of two under the allowable mole fraction estimation error of 3%. To investigate the robustness of C-DCS, we evaluated how the compression rate affects the transmittance, center wavenumber, and linewidth of the reconstructed absorption lines. Finally, we demonstrated the massively parallel sensing of 10 molecular species and showed the compression rate over 10. Our numerical studies show that the C-DCS is promising for solving the foreseeable data size problem in future dual-comb spectrometer deployment.

## Data Availability

The data provided in the manuscript are available from T.I. upon request.
